# Highly sensitive detection of driver mutations from cytological samples and cfDNA in lung cancer

**DOI:** 10.1002/cam4.4330

**Published:** 2021-10-07

**Authors:** Kazutaka Fujita, Masayuki Nakayama, Masafumi Sata, Yoshiaki Nagai, Shu Hisata, Naoko Mato, Takuji Suzuki, Masashi Bando, Nobuyuki Hizawa, Koichi Hagiwara

**Affiliations:** ^1^ Department of Pulmonary Medicine University of Tsukuba Tsukuba Ibaraki Japan; ^2^ Division of Pulmonary Medicine Department of Internal Medicine Jichi Medical University Shimotsuke‐shi Tochigi Japan

**Keywords:** cell‐free DNA, cytological samples, *EGFR* mutation, lung cancer, secondary mutation

## Abstract

**Background:**

Bronchoscopy is a minimally invasive procedure for establishing the diagnosis of lung cancer. It sometimes fails to obtain tissue samples but readily collects cytological samples.

**Methods:**

We developed PNA‐LNA dual‐PCR (PLDP), which amplified mutant sequences by a high‐fidelity DNA polymerase in the presence of a peptide nucleic acid (PNA) oligomer having a wild‐type sequence. Mutations are detected either by locked nucleic acid (LNA) probes for quick detection of a limited number of mutations, which are *EGFR*, *KRAS*, and *BRAF* mutations in the current study, or by direct sequencing for a comprehensive screening. In a total of 233 lung cancer samples, the results for cytological samples by PLDP were compared with those for tissue samples by cobas® *EGFR* mutation test (cobas) or by the PNA‐LNA PCR clamp method (P‐LPC). Moreover, the performance of PLDP using cell‐free DNA (cfDNA) was investigated.

**Results:**

Peptide nucleic acid‐LNA dual‐PCR was able to detect each synthesized mutant sequence with high sensitivity. PLDP detected *EGFR* mutations in 80 out of 149 clinical samples, while the cobas or the P‐LPC detected in 66 matched. The correctness of PLDP was confirmed both by clinical response and by the results of sequencing using a next‐generation sequencer. PLDP detected mutations from cfDNA in approximately 70% of patients who harbors mutations in the tumor.

**Conclusions:**

Peptide nucleic acid‐LNA dual‐PCR exhibited an excellent performance, even using cytological samples. PLDP is applicable for the investigation of cfDNA. The combination of bronchoscopy and PLDP is attractive and will expand the utility of bronchoscopy in clinical practice.

## Graphical abstract

Peptide nucleic acid‐LNA dual‐PCR sensitively detects mutations from cytological samples or cell‐free DNA in lung cancer. It will expand the utility of cytological specimens in clinical settings where bronchoscopy is employed.

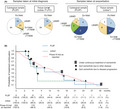

## 1 INTRODUCTION

Bronchoscopy is broadly employed in the diagnosis of lung cancer. Accordingly, most lung cancers are diagnosed by bronchoscopy in Japan, while percutaneous needle biopsy is applied only in limited cases. Furthermore, with technical advances in the endobronchial ultrasound‐guided transbronchial needle aspiration and the endobronchial ultrasonography with guide‐sheath procedures,^1^, ^2^, ^3^ isolation of samples from small peripheral lesions or transmurally from the mediastinal lymph nodes has become possible. Consequently, the size of specimens becomes much smaller and a cytological sample is often the only sample obtained. Cytological samples are sufficient for establishing the diagnosis of lung cancer. A procedure that authenticates cytological samples for mutation testing has been documented.^4^ Nevertheless, most of the mutation tests that have been introduced into clinical practice requires tissue samples. The development of tests that enables the investigation of cytological samples is anticipated.

Oncogenic driver mutations are found in 62–64% of lung adenocarcinoma.^5^, ^6^, ^7^ The frequency rises to 71% in East Asia.^8^ Administration of molecular targeting drugs matching the mutation has demonstrated an excellent clinical response and a decrease in the mortality rate.^9^ The drugs include epidermal growth factor receptor (EGFR)‐tyrosine kinase inhibitors (TKI) for lung cancers with *EGFR*‐sensitive mutations,^10^, ^11^, ^12^, ^13^, ^14^, ^15^, ^16^, ^17^ osimertinib for those with the *EGFR* T790M mutation,^18^ anaplastic lymphoma kinase (ALK) inhibitors for those with *ALK* mutations, and ROS1 inhibitors with c‐ROS oncogene 1 (*ROS‐1*) fusion genes.^19^, ^20^, ^21^, ^22^ Currently, investigation of oncogenic driver mutations has become mandatory in clinical practice.

To harmonize the advanced bronchoscopic procedures with mutation testing, we developed a highly sensitive method, peptide nucleic acid (PNA)^23^, ^24^‐locked nucleic acid (LNA)^25^‐dual PCR (hereafter PLDP), that quickly detects mutations from cytological samples. The reaction completes within 2 h after DNA isolation. First, a high‐fidelity PCR reaction with a PNA clamp primer(s) preferentially amplifies mutant sequences. Then, an LNA‐probe(s) is employed to detect a limited number of frequent mutations. If no mutations are found, direct sequencing is performed for a comprehensive mutation search, which takes one more day. In the current study, *EGFR* exon 19 deletion p.E746_A750del [Catalog of Somatic Mutation in Cancer (https://cancer.sanger.ac.uk/cosmic) mutation ID (COSM) 6223]; *EGFR* exon 19 deletion p.E746_A750delELREA (COSM6225); *EGFR* p.T790M (COSM6240, hereafter *EGFR* T790M); *EGFR* p.L858R (COSM6224, hereafter *EGFR* L858R); *KRAS* p.G12C (COSM516); and *BRAF* p.V600E (COSM476, hereafter *BRAF* V600E) were entitled as frequent mutations and investigated by LNA‐probes. They are able to identify most of the patients to whom current molecular targeting drugs are applicable.^10^, ^11^, ^12^, ^13^, ^14^, ^15^, ^16^, ^26^, ^27^, ^28^ Hereafter, *EGFR* exon 19 deletions p.E746_A750del plus p.E746_A750delELREA are collectively called as Ex19del, *EGFR* p.L858R is as L858R, and *EGFR* p.T790M as T790M.

Cell‐free DNA (cfDNA) isolated from peripheral blood is an alternative sample for mutation testing when both tissue and cytological samples are not available. PLDP may be applicable to cfDNA, which we also investigated.

The PLDP has been in operation since 16 December 2016. Here, cytological samples were investigated by PLDP. When tissue samples were also obtained, they were submitted either to the cobas® *EGFR* mutation test version 2^29^, ^30^ (cobas hereafter) or to the PNA‐LNA PCR clamp (P‐LPC).^31^ Moreover, cfDNA was tested by PLDP. This practice allowed us to test multitypes of samples (a sample set) by multiple tests and compare their performance. The results were as follows: (1) PLDP outperformed the cobas or the P‐LPC; (2) PLDP detected more mutations in cfDNA as the disease stage advances. Thus, PLDP is an excellent option in clinical settings where cytological samples are highly engaged. Although the use of cfDNA does not completely replace the use of cytological samples or tissue samples, it may be an alternative option for patients with advanced diseases.

## 2 MATERIALS AND METHODS

### 2.1 Clinical samples

A sample set consisted of a cytological sample, a tissue sample, and a blood sample. The cytological and tissue samples were collected from the same lesion. The blood sample was collected on the same day. A sample set may lack a tissue sample because a tissue sample is more difficult to collect than a cytological or a blood sample in clinical practice. Cytological and blood samples were tested by PLDP in our laboratory. Tissue samples were formalin‐fixed, paraffin‐embedded, and submitted to the *EGFR* mutation test at the LSI Medience Corporation (Tokyo, Japan) either by the cobas or by the P‐LPC depending on the physicians' preference.

### 2.2 DNA extraction

Cytological samples pathologically confirmed positive for cancer was centrifuged at 1200× g for 3 min (Figure S1A). DNA was extracted from the precipitate using the Promega Maxwell^®^ RSC AS1400 (Promega). Plasma was isolated from blood samples by two times of centrifugation at 260× g for 10 min. DNA was extracted using the Promega Maxwell^®^ RSC AX1114/AX1115 (Figure S1B).

### 2.3 PLDP reaction

**FIGURE 1:**
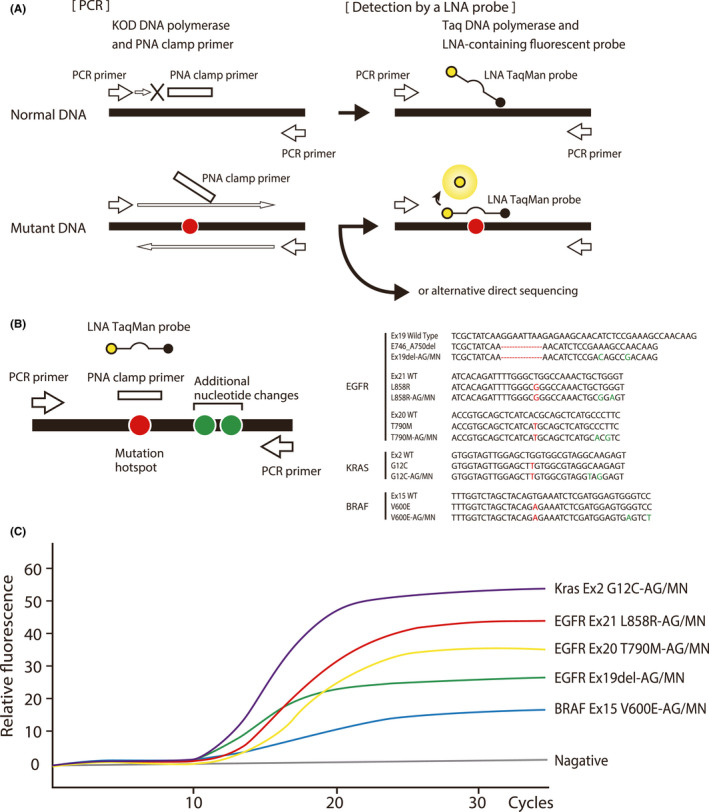
Peptide nucleic acid‐locked nucleic acid dual‐PCR reaction. (A) Reaction. (B) Structure of plasmids that have two additional nucleotide changes that discriminate the plasmid‐derived sequences from the artifacts. (C) The fluorescence curves obtained at the limit of detection (LOD)

Polymerase chain reaction was performed in a 25‐µl reaction containing 10–50 ng DNA, six primer pairs (60 nM for *EGFR* exon 18 mutations and 160 nM for the others), six different PNA clamp primers (4 µM each), 1× KOD buffer #2, 200 nM dNTPs, 1 mM MgSO_4_, and 0.5 units of KOD‐plus‐DNA polymerase version 2 (PCR enzyme and derived from DNA polymerase extracted from bacteria Thermococcus kodakarensis KOD 1 strain) (Toyobo [KOD‐211]; Figure 1A; Figure S2A). The PCR cycling was a 94°C hold for 120 s followed by 45 cycles at 94°C for 6 s, 58°C for 6 s, and 68°C for 30 s. The detection reaction with the LNA‐probe(s) was performed in six separate tubes. Each tube contained a 25‐µl reaction consisted of 1 µl of 1000‐fold diluted PCR product, 200 nM of PCR primers, 100 nM each of LNA‐probe, 1× Ex Taq buffer, 200 nM dNTP, and 0.625 units of Ex Taq DNA polymerase hot start version (Takara Bio [RR006]; Figure S2B,C). The cycling was a 95°C hold for 30 s followed by 35 cycles of 95°C for 15 s and 61°C for 30 s.

In PLDP, the amplification step and the detection step are separated into two PCR reactions. The KOD‐plus‐DNA polymerase version 2, which has 80 times higher‐fidelity than Taq DNA polymerase, provides a large amount of DNA without incorporating artifactual mutations and allows highly sensitive detection of inherent mutations. Thus, PLDP outperforms P‐LPC.

### 2.4 Limit of detection

Plasmid with a mutant sequence and two additional nucleotide replacements were constructed (Figure 1B). The nucleotide replacements marked the sequence as having a plasmid origin. Samples for investigating Limit of detection (LOD) contained 10^3^, 10^2^, 10^1^, or 0 copies of each mutation in 50 ng of normal human genomic DNA (1.5 × 10^4^ copies of a haploid genome).^4^ LOD was determined by investigating 48 samples for each dilution.

### 2.5 Direct sequencing

Direct sequencing was outsourced to FASMAC. The samples were shipped by the postal service and the results were received the next day by e‐mail.

### 2.6 Sequencing using a next‐generation sequencer

Mutations in some of the cytological samples were exploratorily investigated using the MINtS system that employs the MiSeq sequencer (Illumina K.K.) as previously reported.^32^

## 3 RESULTS

### 3.1 System design

A high‐fidelity DNA polymerase, KOD‐plus‐DNA polymerase version 2^33^ (Toyobo), was used for PCR in the presence of a clamp primer that is, an oligomer of PNA having a wild‐type sequence. The clamp primer anneals to the target amplicon, inhibits the amplification of the wild‐type sequence, and preferentially amplifies the mutant sequences. Each mutation was detected by an LNA‐probe that was TaqMan probes^34^ having a LNA at the mutation site (Figure 1A). Other mutant sequences that overlap the PNA oligo sequence were also preferentially amplified over the wild‐type sequence. Amplified DNA fragments where mutations were undetected by the LNA probes were submitted to direct sequencing in search of other mutations.

### 3.2 Limit of detection

In the clinical samples, mutant sequences and the background wild‐type sequences co‐exist. The LOD, which is the lower limit of the frequency of a mutant sequence that a method detects, is one of the essential indicators of its performance. Plasmids with a mutation hotspot sequence, together with two extra nucleotide replacements in the flanking sequence (Ex19del‐AG/MN, T790M‐AG/MN, L858R‐AG/MN, G12C‐AG/MN, or V600E‐AG/MN [AG: artificial gene, MN: maker nucleotide]), were synthesized to determine the LOD (Figure 1B). These nucleotide replacements were for discriminating the sequence from the artifact mutant sequences that stemmed from a DNA polymerase error during PCR reaction.

Peptide nucleic acid‐LNA dual‐PCR reaction detected 10^2^ copies of a mutant sequence in 50 ng genomic DNA (1.5 × 10^4^ copies of haploid genome) for Ex19del‐AG/MN, 10^1^ for T790M‐AG/MN, 10^2^ for L858R‐AG/MN, 10^2^ for G12C‐AG/MN, and 10^2^ for V600E‐AG/MN (Figure 1C). The LOD was 0.0007–0.007. The sensitivity and specificity by investigating 48 samples were both 1.0. No artifactual mutation was detected. The LOD of the P‐LPC, which is one of the most sensitive tests clinically used, was similarly investigated. The results showed that the P‐LPC detected 10^3^ copies for Ex19del‐AG/MN, 10^3^ for T790M‐AG/MN, 10^3^ for L858R‐AG/MN, 10^3^ for G12C‐AG/MN, and 10^4^ for V600E‐AG/MN. The LOD was 0.017–0.17. We concluded that PLDP outperformed the P‐LPC. The LOD of cobas was 0.05 according to the manufacture's information and thus was not tested in the current study.

### 3.3 Clinical samples

Most of the patients enrolled had stage IVA (24%)–IVB (31%) adenocarcinoma or squamous cell carcinoma (Table 1; Table S1). Samples were isolated either at the time of diagnosis or at the time of disease exacerbation. The presence of cancer cells was pathologically confirmed. A total of 233 sample set (149 sets had both a cytological sample and a tissue sample, while 84 had only a cytological sample) and were enrolled from 16 December 2016 to 11 March 2019. Some patients provided samples at both at the time of diagnosis or at the time of disease exacerbation; thus, two sample sets may be enrolled for some patients.

**TABLE 1:** Characteristics of samples

Origin	Bronchoscopy specimen	194
	Pleural effusion	22
	Lymph node aspiration	6
	Others^a^	11
Stage	0	2
(8th RECIST)	IA1–3	36
	IB	5
	IIA–B	21
	IIIA–C	40
	IVA–B	129
Pathological diagnosis	Adenocarcinoma	169
	Squamous cell carcinoma	45
	Non‐small cell lung cancer	7
	Carcinoma	4
	Others^b^	8

^a^ Percutaneous needle aspiration, sputum, pericardial fluid, echo‐guided percutaneous liver needle biopsy, cerebrospinal fluid, and echo‐guided lymph node needle biopsy obtained by gastroscopy.

^b^ Adeno‐squamous carcinoma, neuroendocrine cell carcinoma, ciliated muconodular papillary tumor, and large cell neuroendocrine carcinoma plus squamous cell carcinoma.

### 3.4 Detection of EGFR by PLDP in clinical samples

**FIGURE 2:**
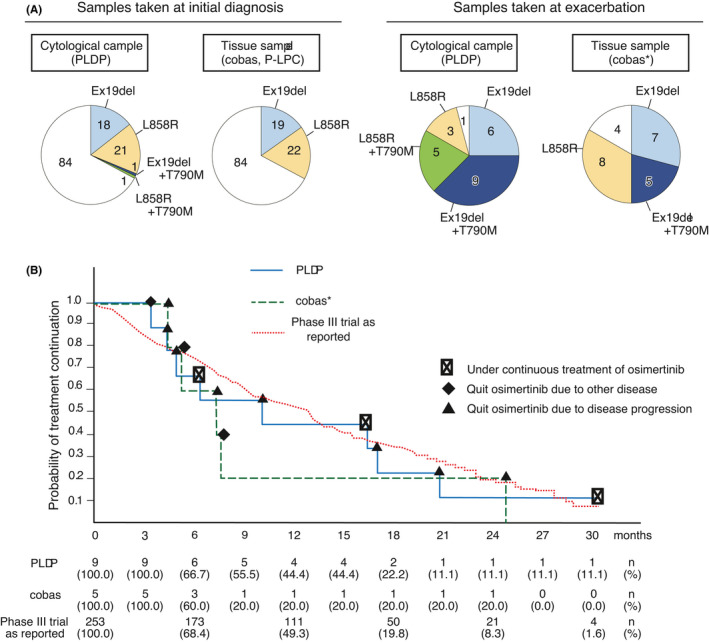
Clinical performance of peptide nucleic acid (PNA)‐locked nucleic acid (LNA) dual‐PCR (PLDP). (A) Mutations detected using cytological samples by PLDP and tissue samples by cobas *EGFR* mutation test or PNA‐LNA PCR clamp method (P‐LPC). Sample sets that had both cytological samples and tissue samples were investigated. A total of 43 mutations (Ex19del, 19; L858R, 22; and T790M, 2) were detected from cytological samples in combination with PLDP, and that of 41 mutations (Ex19del, 19; L858R, 22; and T790M, 0) were detected from tissue samples in combination with cobas or P‐LPC at initial diagnosis. In addition, a total of 37 mutations (Ex19del, 15; L858R, 8; and T790M, 14) was detected from cytological samples in combination with PLDP, and that of 25 mutations (Ex19del, 12; L858R, 8; and T790M, 5) were detected from tissue samples in combination with cobas at exacerbation. (B) Time to treatment failure survival curve. All samples positive for T790M by cobas were also positive by PLDP, thus PLDP detected additional patients to whom osimertinib was effective. The result of a randomized phase III clinical trial is shown overlaid. *We found physicians only used cobas for samples taken at exacerbation and thus no data were available for P‐LPC

Cytological samples were obtained by bronchoscopy (84%), obtained as pleural effusion (9%), or obtained by the other procedures (7%) (Table 1). A total of 149 sample sets had both cytological samples and tissue samples. Thus, we were able to compare the results between them. We analyzed *EGFR* mutation in cytological samples tested by PLDP and in tissue samples tested by cobas or P‐LPC. A total of 80 mutations (Ex19del, 34; L858R, 30; and T790M, 16) were detected from cytological samples, and 66 (31, 30, and 5) from tissue samples. We subgrouped the samples into those obtained at initial diagnosis and those obtained at exacerbation. This is because re‐biopsy at exacerbation is a common clinical practice for adenocarcinoma in search of T790M mutation for which osimertinib is effective (Figure 2A). The results for samples obtained at initial diagnosis well concorded, indicating that cytological samples are excellent material for mutation tests. On the other hand, sample sets taken at exacerbation showed discordant results. Here, PLDP detected T790M in more samples: cobas detected T790M in a fraction of samples in which PLDP detected T790M. The samples taken at exacerbation may contain more fibrous tissue and harbor more heterogeneous cell populations, making mutation detection more difficult. We speculated that better sensitivity of PLDP contributed to a higher detection rate. To confirm the speculation, we performed two investigation. First, we submitted DNA to a next‐generation sequencer‐based system, MINtS.^32^ The rate of T790M detected was cobas < MINtS = PLDP. Second, we retrospectively investigated the survival curve of the median time to treatment failure (Figure 2B). Here, one plot is for five patients who were administered osimertinib because cobas detected T790M. PLDP detected all these five patients and four more patients with T790M. Thus, the other plot is for a total of nine patients who were administered according to the results by PLDP. Although a statistical analysis may be difficult because of a limited number of patients, patients selected by PLDP seems to show a plausible response. We concluded that, at least for T790M mutation, an excellent performance of PLDP contributed to better detection.

### 3.5 Results of cytological samples and cfDNA

**FIGURE 3:**
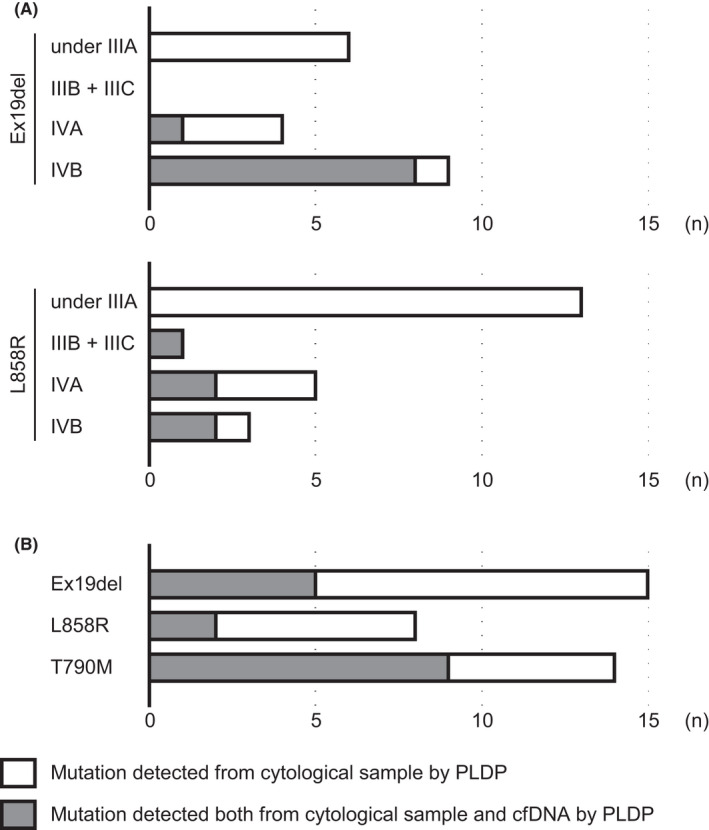
Performance of peptide nucleic acid (PNA)‐locked nucleic acid (LNA) dual‐PCR (PLDP) on cfDNA. (A) The number of mutation‐positive samples at initial diagnosis were shown by stage. The detection of Ex19del from cfDNA/cytological samples was 0/6 under IIIA, 0/0 in IIIB+IIIC, 1/4 in IVA, and 8/9 in IVB. The detection of L858R from cfDNA/cytological samples was 0/13 under IIIA, 1/1 in IIIB+IIIC, 2/5 in IVA, and 2/3 in IVB. (B) The number of mutation‐positive samples at exacerbation. The detection of Ex19del, L858R, and T790M from cfDNA/cytological samples was 5/15, 2/8, and 9/14

We compared the frequency of *EGFR* mutation detected from cytological samples and cfDNA using 149 sample sets. PLDP detected mutations in 80 cytological samples (Ex19del, 34; L858R, 30; and T790M, 16) and in 30 cfDNA (Ex19del, 14; L858R, 7; and T790M, 9) (Figures 2A and 3). The frequencies were comparable with previous studies.^35^, ^36^, ^37^, ^38^, ^39^, ^40^ These indicate that cfDNA may provide false‐negative results in a significant proportion of the patients even using highly sensitive methods like PLDP.

## 4 DISCUSSION

Detection of oncogenic driver mutations from a small sample is important in clinical practice. Nevertheless, many of the mutation tests having introduced into clinical medicine require a large tissue sample and often analyze genes for which no drugs are currently available on the market. There is an apparent discrepancy between the specification of these tests and the clinical needs. One of the reasons we developed PLDP is to fill this gap.

Peptide nucleic acid‐LNA dual‐PCR (LOD: 0.0007–0.007) exhibited a good performance in comparison with cobas (LOD: 0.05, according to the instruction sheet) or the P‐LPC (LOD: 0.017–0.17). PLDP detected 10^1^–10^2^ copies of mutant sequences in 50 ng of human genomic DNA (1.5 × 10^4^ copies); this is the rate comparable to one mutant cell to 1000 normal cells. The cancer cell content of cytologically cancer‐positive samples is usually >1%.^41^ Furthermore, 10–50 ng of DNA is readily available from cytological samples isolated by bronchoscopy. This indicates that PLDP has specifications suitable for application to clinical settings where bronchoscopy is highly engaged. Previous reports^41^, ^42^, ^43^ have demonstrated that the utility of cytological samples in combination with P‐LPC for mutation detection. However, they lacked to investigate the LOD, and thus the utility of P‐LPC to more challenging materials including samples with small number of cancer cells or cfDNA was hard to speculate. In the current study, we clarified the performance and the LOD of PLDP and both were excellent. PLDP may serve as a good touch stone for evaluating novel mutation tests.

The list of LNA‐probe(s) used in this study detects only a limited number of mutations. Nevertheless, it covers 75% of the occurrences of the drug‐sensitive *EGFR* mutations. Moreover, PNA clamp primer was designed to preferentially amplify many of the other *EGFR* mutations. As a result, 90% of the drug‐sensitive *EGFR* mutation occurrences are detected with a use of the direct sequence step.^44^, ^45^ The insurance system may require mutation testing by approved companion diagnostics before the use of corresponding drugs. Even in such cases, prescreening using PLDP provides valuable information, and physicians will be prepared for the treatment before the result of companion diagnostics is returned.

Secondary mutations that occur during the treatment of molecular targeting drugs are a cause of drug resistance. They are known to occur in a limited number of sites. Nevertheless, their detection is often difficult because the tumor contains more amount of connective tissue and the cells are more genetically heterogeneous than at the initial treatment.^46^, ^47^ Accordingly, a highly sensitive mutation test is required. PLDP is suited to meet this need, as shown in the detection of T790M in the current study.

One of the principal clinical questions is whether cfDNA serves as an alternative of cytological or tissue samples in mutation testing. We preliminary investigated *KRAS* codons 12 and 13 mutations and found the detection rate was similar to that obtained for *EGFR* mutation (Figure S3). The frequencies were comparable with the studies that mainly investigated Stage IV patients^35^, ^36^, ^37^, ^38^ or that investigated patients with acquired EGFR‐TKI resistance.^39^ The source of cfDNA is apoptotic or necrotic cells and may be live cells.^48^, ^49^ The amount depends on the location, size, and vascularity of the tumor, thus it is uncertain whether a patient provide sufficient amount of cfDNA for mutation testing. We found that mutation is sometimes not detected in cfDNA in Stage IV disease and often not detected in Stages IIIA or earlier disease. Thus, cfDNA may not be a good material for diagnosing mutations in the patients with Stages IIIA or earlier disease.

In the current study, we developed a highly sensitive mutation test called PLDP. The combination of PLDP and cytological samples exhibited an excellent performance and considered useful in the clinical settings where cytological samples play an important role. cfDNA from patients with stage IIIB or more disease may partially serve as a material for testing mutation. The current study warrants further investigation on the utility of cytological samples in mutation testing, as our ongoing study in which the utility of the cytology samples is being investigated using a next‐generation sequencer (Clinical trial ID: UMIN000015665 and UMIN000040415).

## ETHICS STATEMENT

The ethical committee of the Jichi Medical University approved the study (IDEN 17‐Rev32). All patients provided written informed consent.

## CONFLICT OF INTEREST

The authors declare no conflict of interest.

## Supporting information

Fig S1

Fig S2

Fig S3

Table S1

## Data Availability

The data that support the findings of this study are available from the corresponding author upon reasonable request.
