# Supplementary figures and images for: Highly sensitive detection of driver mutations from cytological samples and cfDNA in lung cancer
Source: Cancer Med. 2021 Oct 7;10(23):8595–603. doi: 10.1002/cam4.4330 (PMC8633228; doi:10.1002/cam4.4330)

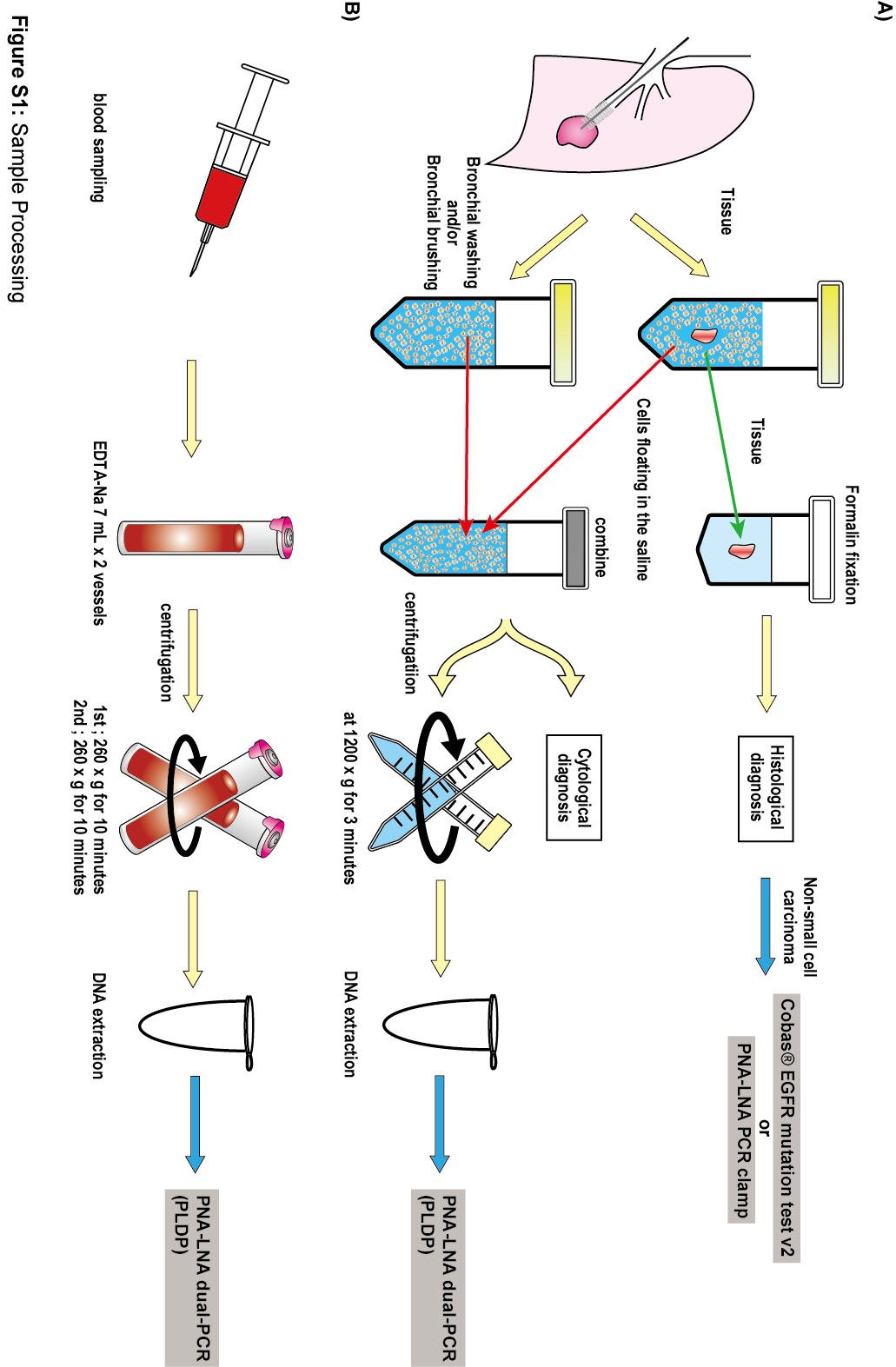

Supplement: Supplementary file 1 — Fig S1 [file CAM4-10-8595-s003.docx]
