# Supplementary material for: Highly sensitive detection of driver mutations from cytological samples and cfDNA in lung cancer
Source: Cancer Med. 2021 Oct 7;10(23):8595–603. doi: 10.1002/cam4.4330 (PMC8633228; doi:10.1002/cam4.4330)
Supplement: Supplementary file 3 — Fig S3 [file CAM4-10-8595-s004.docx]

**Figure S3**: The detection of KRAS mutation

We compared PLDP with MINtS because there was no detection test for KRAS mutations when we started this trial. PLDP detected the mutation in all cases in which MINtS detected the mutation. When the detection of the mutation from cfDNA was compared with that of cytological samples, PLDP detected 8/9 samples in Stages IIIB or more progressive disease (the detection of KRAS mutation from cfDNA/cytological samples was 0/8 under IIIA, 1/1 in IIIB+IIIC, 3/3 in IVA, and 4/5 in IVB).
