# Supplementary material for: Highly sensitive detection of driver mutations from cytological samples and cfDNA in lung cancer
Source: Cancer Med. 2021 Oct 7;10(23):8595–603. doi: 10.1002/cam4.4330 (PMC8633228; doi:10.1002/cam4.4330)
Supplement: Supplementary file 4 — Table S1 [file CAM4-10-8595-s001.docx]

**Table S1**: Characters

|  | Male | | Female | |
| --- | --- | --- | --- | --- |
| Sex | 156 |  | 77 |  |
|  |  |  |  |  |
|  |  |  |  |  |
| Age, years | 67.6 |  | 66.2 |  |
|  | (8.7, 37-85) |  | (10.0, 35-83) |  |
|  |  |  |  |  |
|  |  |  |  |  |
| Smoking status | never | 10 | never | 56 |
|  | NA | 1 |  |  |
|  | ever smokers | 145 | ever smokers | 21 |
|  | 49.9 (31.2, 0.2-225) | | 30.4 (14.8, 5-58) | |

The average (standard deviation, range) is showed in Age.

The lowest line in Smoking status shows the average (standard deviation, range).

The score is displayed as a smoking index, pack-years.
