# Supplementary material for: Highly sensitive detection of driver mutations from cytological samples and cfDNA in lung cancer
Source: Cancer Med. 2021 Oct 7;10(23):8595–603. doi: 10.1002/cam4.4330 (PMC8633228; doi:10.1002/cam4.4330)
Supplement: Supplementary file 4 [file CAM4-10-8595-s002.docx]

Figure S2: PLDP reaction

A

PCR reaction mixture

Reagent	Sequence	Volume	Final concentration
EGFR -exon 18-F*	5'-CTTACACCCAGTGGAGAAGCTC-3'		60 nM
EGFR -exon 18-B	5'-CCCACCAGACCATGAGAGG-3'		60 nM
EGFR -exon 19-F	5'-CTGGTAACATCCACCCAGATCAC-3'		160 nM
EGFR -exon 19-B	5'-CCACACAGCAAAGCAGAAACTCAC-3'		160 nM
EGFR -exon 20-F	5'-CATGCGTCTTCACCTGGAAGG-3'		160 nM
EGFR -exon 20-B	5'-CACACCAGTTGAGCAGGTACTG-3'		160 nM
EGFR -exon 21-F	5'-GCAGAGCTTCTTCCCATGATGATC-3'		160 nM
EGFR -exon 21-B	5'-CCTTACTTTGCCTCCTTCTGCA-3'		160 nM
KRA S-exon 2-F	5'-AACTTGTGGTAGTTGGAGCT-3'		160 nM
KRAS -exon 2-B	5'-CAAGATTTACCTCTATTGTTGGATC-3'		160 nM
BRAF -exon 15-F	5'-CTTCATAATGCTTGCTCTGATAGG-3'		160 nM
BRAF -exon 15-B	5'-GGATCCAGACAACTGTTCAAAC-3'		160 nM
EGFR Exon 19delcl#	NH_2-GATGTTGCTTCTCTTAAT-$CONH_2$		4 µM
EGFR Exon 20cl	NH_2-CTCATCACGCAGCTCA-$CONH_2$		4 µM
EGFR Exon 21cl	NH_2-CAGTTTGGCCAGCCCA-$CONH_2$		4 µM
KRAS -exon 2cl	NH_2-ACGCCACCAGCTCC-$CONH_2$		4 µM
BRAF -exon 15 V600cl	NH_2-AGATTTCACTGTAGCTAG-$CONH_2$		4 µM
Human Genomic DNA			10–50 ng
KOD buffer #2			1×
dNTPs			200 µM
$MgSO_4$			1 mM
KOD plus DNA polymerase version 2			0.5 U
		25 µL	

* Primers with a postfix '-F' and '-B' are oligonucleotides.
# Primers with a postfix 'cl' are peptide nucleic acids.

B

Reaction for the detection of EGFR E746_A750 del type1 by LNA probe

Primer name	Primer sequence	Volume	Final concentration
EGFR -exon 19-F*	5'-CTGGTAACATCCACCCAGATCAC-3'		200 nM
EGFR -exon 19-B*	5'-CCACACAGCAAAGCAGAAACTCAC-3'		200 nM
EGFR E746_A750del type1 - LNA*	6-FAM/CGCTATCAA+AACATCTCCG/BHQ1		100 nM
1000×diluted PCR product		1 µL	
Ex Taq Buffer (Mg^{2+} plus)			1×
dNTP Mixture			200 µM
TaKaRa Ex Taq HS			0.625 U
		25 µL	

* A primer with a postfix '-LNA' are a detection probe containing LNA. The mixture contains primers for the target amplicon and LNA probe for the mutation. The reaction for mutation detection by LNA probe was set up for each mutation, as shown in panel C.

C

Primers and mutation-specific LNA primers for each mutation	Primers for the amplicon	
Mutation	Primers for thhe amplicon	Mutation-specific LNA primer
EGFR exon 19 E746_A750 del type1	EGFR-exon 19-F and -B	6-FAM/CGCTATCAA+AACATCTCCG/BHQ[illegible]
EGFR E746_A750 del type2	EGFR-exon 19-F and -B	6-FAM/CGCTATCAA+GACATCTCCG/BHQ
EGFR Exon20 T790M	EGFR-exon 20-F and -B	6-FAM/ATGAGCTGC+ATGATGA/BHQ1
EGFR Exon21 L858R	EGFR-exon 21-F and -B	6-FAM/TTTGGCC+CGCCCAA/BHQ1
KRAS Exon2 G12C	KRAS-exon 21-F and -B	6-FAM/ACGCCAC+AAGCTCCA/BHQ1
BRAF Exon15 V600E	BRAF-exon 15-F and -B	6-FAM/AGATTT+C+TCTGT+AGC/BHQ1

A "+" in the LNA primer sequence indicates the subsequent nucleotide is a locked nucleic acid. FAM: 5(6)-Carboxyfluorescein. BHQ: black hole quencher.
